# Institutional Needs

**Published:** 1914-10-10

**Authors:** 


### INSTITUTIONAL NEEDS.

### PARABOLIC REFLECTOR LAMPS.

The problem of lighting emergency hospitals is one which has been recently alluded to in The Hospital, and in this connection considerable interest attaches to Bruce's Safety Parabolic Reflector Lamps, which are specially designed for hospital and medical use wherever gas or electric light is not available. Many hospitals already possess them for supplementary purposes, and their chief points are as follows: The oil reservoir is made of English brass, and fitted with a safety tube, a carefully designed improvement. The lamp can be filled by means of a screw filler without the inconvenience and, indeed, the element of risk caused by first removing the burner and glass. The Parabolic reflector both acts as a shade to the eyesight, and also directs a clear white light in whatever direction is required. The reflectors are designed, by the hard metal of which they are constructed, to wear without going black through the heat, and can also be obtained nickel-plated. Special wicks and fireproof glasses combine to make this lamp serve the triple purpose of providing a safe and effective light for ward or duty room, a useful adjunct to the surgery for examination purposes, and a pedestal which can be used as a source of illumination for any purpose for which gas or electric light when available are employed. F. and J. Bruce, of 232 Borough High Street, London, also design lamps for general purposes in all shapes, and of every kind, both standing or hanging.

### HAND-PROPELLED TRICYCLES.

As that of an invalid-chair specialist, whose designs are worth study, the booklet issued by Jas. P. Witham, on "Efficiency in Hand-propelled Invalid Tricycles," is of interest to all who realise how much care is needed in the design of these additional "limbs." His principle is that of lever action and the changeable gear, which last is specially designed for hand-tricycle use. The reasons for this attitude, and the experience on which it is based, make both the letterpress and the illustrations of more than passing interest. The questions of gradients and of avoidance of exertion are gone into: hill-climbing gears are described, and the achievements of various invalids—"forty-five miles in five hours' time, with the help of his machine," is an example—are set forth to the interest of every doctor, nurse, or invalid who turns to its pages. Indeed, a copy will be sent to any Society connected with the sick, medical practitioner, trained nurse, or patient who applies to J. P. Witham, at Pyle House, Newport, Isle of Wight. The author, himself quite unable to stand, began to design machines to help him to get about independently, and that is why there are many touches of insight and experience in this remarkable pamphlet, which we cordially recommend to all readers, whether directly concerned with cripples or not.

### "AQUAPERIA."

The revival of British spas, which has been a feature of recent years, has been naturally accompanied by the introduction of British waters. "Aquaperia" is one of these, and is prepared by Camwal, Ltd., whose intention is understood to be the introduction of a British aperient tonic water with a view to replacing mineral waters from Germany. "Aquaperia" is prepared and bottled at Camwal's spring at Harrogate, though their other factories also supply it, and wholesale prices are offered to hospitals and institutions. It will be strange indeed if the present war does not focus attention upon British products, and "Aquaperia" and the same firm's table-water called "Fontalis" may be commended for a trial on this ground. The Harrogate springs have a reputation which acquaintance can only confirm.

### SHAVING WITHOUT SOAP.

To shave without employing soap, water, or brush, omitting everything, in fact, except the razor, is a feat which can be accomplished by the use of a preparation known as A. S. Lloyd's "Euxesis." The preparation itself is a demulcent cream, moist but not thin in quality, which is applied out of a tube by the finger to the face. No lather, in the accepted sense of the term, results, but the skin is none the less ready for the razor, for while leaving the skin soft after use, it aids the removal of the beard. Its claim to attention is twofold: As a cream which keeps the skin soft after shaving, and as a device for avoiding the multiple apparatus of brush, hot water, and soap. On a journey and to travellers generally a device for shaving which does away with these is sure of a welcome, and what is useful in travelling—by saving trouble—is useful also at home. "Euxesis" is manufactured by Aimée Lloyd and Co., 23 Panton Street, Leicester Square, and is sold in pliable tubes, price 1s. 6d. and 3s. each.

### "EUREKA" HOT-WATER BOTTLES.

The transport of sick and wounded and the comforts provided for them serve to remind us, apart from the season, of the value of the improved type of rubber hot-water bottle. Though the old stone bottle has its uses, it lacks entirely the adaptability, lightness, and unpleasantness when cold of the up-to-date rubber pattern. Indeed, it is by comparing the two that we realise how great is the improvement due to the use of rubber bottles for this purpose. But it is essential that the rubber should be of the best and the workmanship of the highest order, otherwise leakage and all the horrors of an unsatisfactory bottle will ensue. The "Eureka" brand, manufactured by Vincent Wood, of 4 Albion Place, Blackfriars Bridge, S.E., is guaranteed, and may be commended for quality. The favourite size, 12 by 8, with cover of lamb's wool, can be obtained price 6s., and other sizes are priced in proportion.

### EMERGENCY HOSPITAL FURNITURE.

Aseptic hospital furniture, sterilisers, and equipment of every description are manufactured by Messrs. White and Wright, of 93 Renshaw Street, Liverpool, and give them deservedly the name of specialists. While many hospital officers make a point of inspecting the equipment of other institutions, they do not all realise that on occasion time can be as well, if not better, spent in examining the showrooms of certain manufacturers. Where these are properly conducted they give the trained visitor all the advantages of an exhibition with none of the attendant discomforts of crush and crowd. Messrs. White and Wright were one of the first important firms to realise how a show-room should differ from a shop, and claim to-day that their galleries are the very first in the provinces. A fact like this is worth mentioning at the present time when so many emergency hospitals are being equipped, and their new rooms at 93 Renshaw Street, Liverpool, can be commended to all who wish to examine for themselves an extensive stock of every type of hospital equipment.

### THE SKEFFINGTON SPECIALTIES.

49 Ulundi Road, Blackheath, S.E.

Among the ingenious contrivances which have made the reputation of Skeffington's patent lifters, what may be termed an "*x*-ray cushion" is one of the points that attract attention in his recent catalogue. As a patent bed-pan lifting cushion, inflated by a pump, and made in such a way that when fully inflated its ends cannot expand more than the middle, its value is obvious, but it is recommended also for its use as a lifter in connection with *x*-ray purposes, on the ground that the rays are found to pass through it practically unimpeded. Again, the inclinator, for tilting bedsteads without difficulty, presents points that will appeal at once to those experienced in treating and nursing cases requiring a high inclination. Lightness and strength are its qualities, which mean in practice that it is easy to move and attach. Moreover, it can be hired or bought, as required. The Sacrum Lifter is also worth study. No description can do adequate justice to the way in which mechanical aid has been thought out and applied here. Illustrations give some idea, but a practical test is the best testimony to its usefulness. As a labour-saving device, and as enabling one person to do easily what two skilled persons would find difficult, it is remarkable. The "Anastasia" Laminectomy Lifter is a more comprehensive device, and is designed to serve all the purposes of the "Skeffington," the "Skeffington Inclinator," the "Sacrum Lifter," and the "Skeffington Collapsible Sectional Mattress." Here especially a careful study of the illustrations of the apparatus will reveal how a seemingly complex arrangement resolves itself into a series of simple devices for effecting certain desirable movements of patients, which human arms and hands cannot always be spared or be found to perform. A ward can be served by the "Anastasia," which is really an economy in labour and a comfort to patient and nurse alike.

### PATRIOTIC FIRMS.

Among those firms who cater for institutional needs and the sick is the Oxo Company, which has notified the members of its various staffs throughout the country that full pay will be granted to all Territorials and Reservists in its employ who are called to the Colours. All positions will be kept open.

### "MICROBENE."

The need for a fluid cleansing antiseptic is too well known to be insisted on, except possibly in some of those emergency hospitals or adapted houses which are now in process of transformation. A typical example of such a saponaceous fluid is that known as "Microbene," which is manufactured by Robert Young and Co., Ltd., 38 Elliott Street, Glasgow, and is employed in many institutions, such as those devoted to maternity work, where an antiseptic of this character is necessarily extensively used. It is to be noted that "Microbene" is not a coal-tar disinfectant, but a fluid which forms a clear solution with water. It is designed to disinfect and make surgically clean instruments placed in it, and is also stated to be of equal value in the washing of hands and wounds. The makers have branches at 17 St. Ann's Square, Manchester, and 90 Cannon Street, London, and "Microbene" is obtainable in bottles, the largest of which contains 16 oz., and also in gallon and half-gallon tins.

### ANTISEPTIC DUSTING-POWDER.

E. T. Pearson and Co., Ltd., whose offices are at 49 Watling Street, have placed on the market an antiseptic dusting-powder, known as "Peralline Toilet Powder," designed to relieve inflammatory conditions, and to act as a deodoriser and absorbent. Its constituents are stated to be talc, boric acid, and peroxide, whose effect is to produce nascent oxygen upon coming into contact with secretions of the skin. In the sick-room it may be used as an antiseptic dusting-powder in the treatment of wounds, and can be employed on infants' skins after bathing and for preventing soreness in nursing mothers. On the other hand, for everyday use a trial should certainly be made of its qualities, whether as a toilet preparation proper after shaving, or for blistered feet due to vigorous exercise. It is sold in patent tins, price sevenpence, and, as a scientific preparation, is equally serviceable for institutional and for private purposes and for the use of hospital staffs and patients.

### "AZOULE" TUBES OF STERILISED SUTURES.

Messrs. Allen and Hanbury have devoted great attention to the perfection of a method of sterilising catgut, and the difficulties which have hitherto proved almost insurmountable are now apparently overcome. Most severe tests have been passed by material which has been subjected to the special process which this firm have employed, and which depends entirely upon heat and not on chemical methods. The "Azoule" catgut and other sutures (silk, horsehair, silkworm gut, kangaroo tendon, etc.) are put up in breakable glass tubes, the contents of which are designated by plain and indelible inscriptions on the outside instead of by the usual paper label inserted inside the tube. We have carefully examined some of these sutures, and have gathered a most favourable impression both of their material and their reliability. The method in which they are stored in glass tubes—some with a needle already threaded for use—is one calculated to be of great service, safe, and most convenient for emergency work. The catgut is prepared in all sizes, and may be obtained plain, iodised, tanned iodised, and chromicised. The price of the tubes of any sutures, including the emergency ones with needle, is 9d. Very fine black silk has also been prepared for the purpose of suturing blood-vessels.

### RONUK.

Now that the question as to the share of Germans in our industrial enterprises is being discussed it is satisfactory to come across an undertaking like the Ronuk (Floor Polish) Company. All the shares, we are informed, are held by Britishers, and the board and the whole of the staff are the same.

### "ACROSYL."

Mr. V. H. Graesser writes from Ruabon: "I have seen your notice re 'Acrosyl,' and in view of the statement that this article 'is said to be manufactured in England,' it may be of interest to you to learn that I am the manufacturer of this product throughout. My firm is, as you are doubtless aware, a very large producer of cresols and carbolic acid, and I am in a position to supply practically unlimited quantities of 'Acrosyl.' In order to get the name more widely known we are selling same under the name of The Acrosyl Co., London. I may state my works have been established at Ruabon, North Wales, since 1867. . . . No foreigners are employed."

**Owing to the pressure upon our columns this week, we have been compelled to omit "The Institutional Worker" Supplement. This feature, however, will be resumed with our next issue, October 17.**

